# Editorial: MicroRNA: the swift development in infectious diseases

**DOI:** 10.3389/fmicb.2025.1590218

**Published:** 2025-03-25

**Authors:** Sabreena Safuan, Siddhartha Pati, Hisham Atan Edinur

**Affiliations:** ^1^School of Health Sciences, Universiti Sains Malaysia, Health Campus, Kota Bharu, Kelantan, Malaysia; ^2^NatNov Bioscience Private Limited, Balasore, India

**Keywords:** microRNA, infectious diseases, vector-borne diseases, zoonotic disease, treatments

MicroRNAs (miRNAs) are short, non-protein-coding ribonucleic acid (RNA) molecules that act as post-transcriptional regulators via binding to the 3′ untranslated regions (3′-UTRs) of target messenger RNAs (mRNAs) (Ratti et al., 2020). They negatively regulate gene expression, particularly by degrading target mRNAs or inhibiting the translation of proteins. Thousands of them have been well characterized and shown to act on cell growth signaling pathways, differentiation, apoptosis, pathogen-host interactions, stress responses, and immune function (Tribolet et al., 2020; Kimura et al., 2023). The discovery of miRNAs as important players in gene regulation has opened up a new dimension of research in molecular biology, particularly in the study of infectious diseases. Therefore, this Research Topic aims to collect the latest advancements on the impact of miRNAs on pathogen-host interactions and their potential as prognostic biomarkers and therapeutic targets.

During infection, significant changes in miRNA expression can be detected as part of the immune response modulation to protect the host. Pathogens can also exploit the host's miRNA machinery to evade detection, suppresses immune responses, and facilitate colonization within the host. In this Research Topic, Bostanghadiri et al. investigated the association between *Fusobacterium nucleatum*, a Gram-negative anaerobic bacterium, and miRNA expression levels in colorectal adenocarcinoma and matched adjacent normal tissues. They reported higher levels of *F. nucleatum* and elevated expression of cytokines (IL-6, IL-17, TNF-α, and TLR-4) in tumor tissues compared to normal tissues. miR-21 and miR-31 gene expression showed significant fold change in cancer vs. normal tissue, with the former miRNA correlated with high concentrations of *F. nucleatum*. Their findings suggested that miR-21 could be a potential biomarker of colorectal cancer. Wang et al. also discussed the exploitation of host miRNA expression by *Echinococcus* parasites in the immune evasion process. Their study investigated the immunomodulatory mechanisms of mesenchymal stem cells (MSCs) treated with hydatid antigens isolated from sheep and mice. The functional and expression analyses demonstrated the effects of hydatid antigens on miR-146a and miR-9-5p levels in the extracellular vesicles (EVs) extracted from the MSCs.

miRNAs as prognostic biomarkers and therapeutic targets were also discussed in detail in this Research Topic. Muchtar et al. reported microRNA-3145 as a potential therapeutic tool for hepatitis B virus treatment. miR-3145 inhibits HPV replication by downregulating hepatitis B virus S antigen and hepatitis B virus X. In a separate study, Jin et al. conducted a meta-analysis to evaluate the value of miRNAs as both prognostic and diagnostic biomarkers in sepsis patients. A total of 55 studies were included in the meta-analysis, with miR-133a-3p showing the highest diagnostic accuracy. Other miRNAs, including miR-146a, miR-21, miR-210, miR-223-3p, and miR-155, could also potentially be used as biomarkers for predicting sepsis mortality. In another review, Venkatesan et al. discussed the potential applications of exosome miRNAs as diagnosis markers of vector-borne diseases. These include for malaria, schistosomiasis, rickettsia, Chagas, and Japanese encephalitis. They also highlighted the lack of plasma exosome research, although plasma-derived exosome miRNAs were shown to be differentially expressed in vector-borne infected hosts.

In conclusion, the articles collected under this Research Topic provide important updates on the role of miRNAs during pathogen colonization and disease pathogenesis. Understanding miRNA biogenesis and the associated machinery in the context of infectious diseases is not only essential for advancing our knowledge but also carries significant potential for the development of novel prognostic biomarkers and therapeutic strategies.
